# Supplementary material for: Interplay between particle size and microbial ecology in the gut microbiome
Source: ISME J. 2024 Aug 30;18(1):wrae168. doi: 10.1093/ismejo/wrae168 (PMC11406467; doi:10.1093/ismejo/wrae168)
Supplement: ms_particleSize_supp_revision2_CLEAN_08262024_wrae168 [file ms_particlesize_supp_revision2_clean_08262024_wrae168.docx]

**Supplementary Information for**

Interplay between particle size and microbial ecology in the gut microbiome

Jeffrey Letourneau, Verónica M Carrion, Jun Zeng, Sharon Jiang, Olivia W Osborne, Zachary C Holmes, Aiden Fox, Piper Epstein, Chin Yee Tan, Michelle Kirtley, Neeraj K Surana, and Lawrence A David

Corresponding author: Lawrence A David

**Email:** [lawrence.david@duke.edu](mailto:lawrence.david@duke.edu)

**This file includes:**

Figures S1 to S16

| 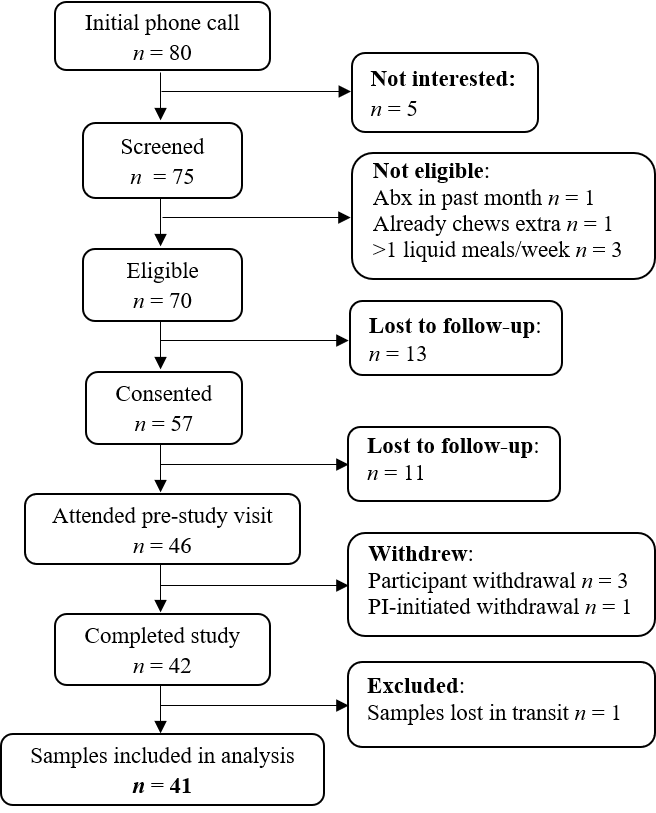 |
| --- |
| **Figure S1. Recruitment and enrollment statistics for Cohort 1.**  Analytic sample size flowchart in the style of Strengthening The Organization and Reporting of Microbiome Studies (STORMS). An equivalent diagram for Cohort 2 was previously published^35^. |


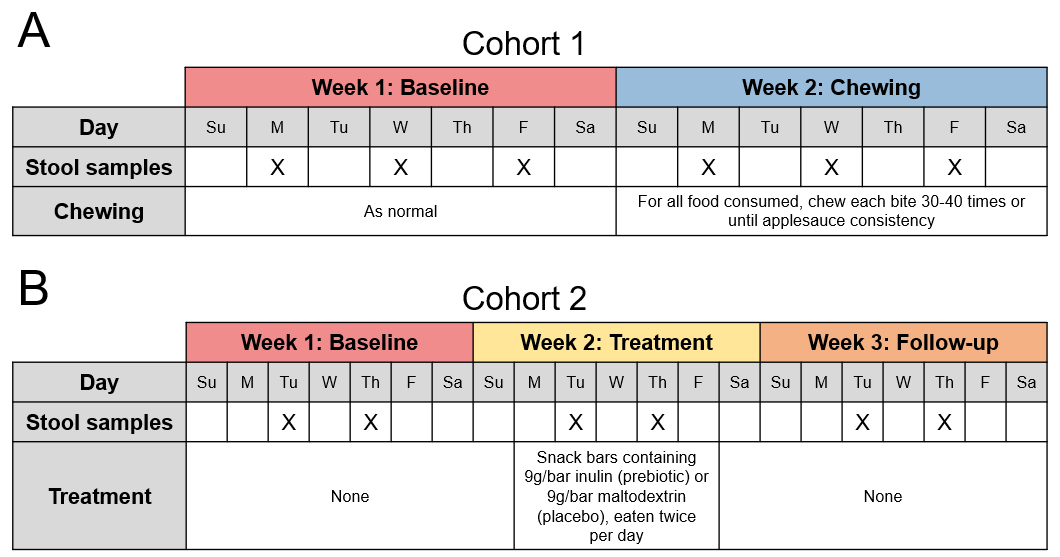


**Figure S2.** **Original study designs for the two cohorts.**

**A**, Cohort 1 (*n* = 41) was recruited specifically to study the role of particle size in the gut microbiome. This study consisted of two weeks, with three stool sample collection points per week. For Week 2, participants were instructed to continue eating their usual diet, with the sole change of chewing each bite of all food consumed approximately 30-40 times or until the food reached an applesauce consistency. **B**, Data from Cohort 2 constitutes a *post hoc* analysis of samples left over from a previously described cohort (original *n* = 40; samples remaining for use in this follow-up analysis *n* = 35). The original study design was three weeks, with two stool samples per week. However, to avoid effects of treatment influencing our investigation into particle size dynamics, only samples from the Baseline week were analyzed in the present study.


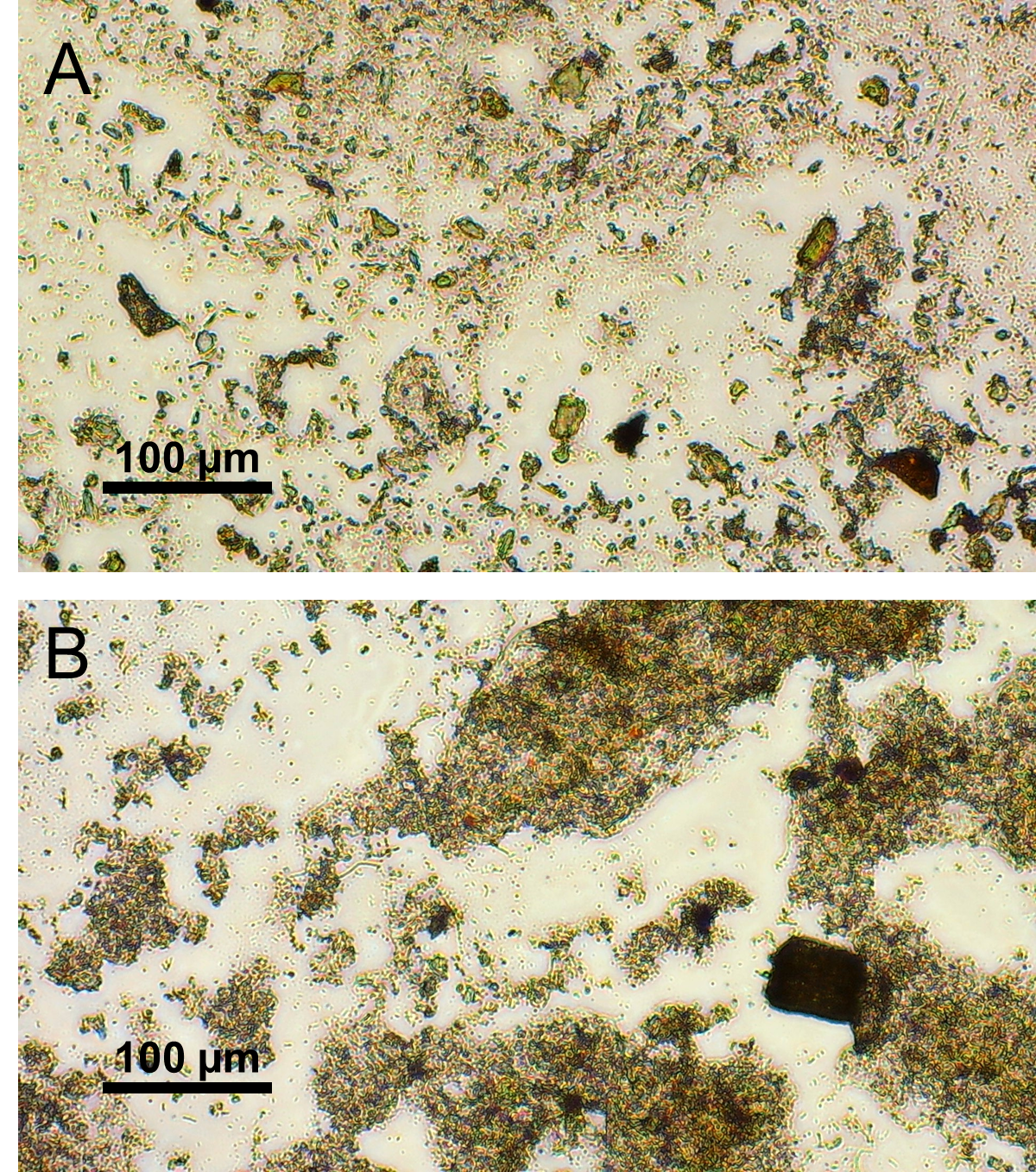


**Figure S3. Microscopy image examples of small and large fecal particles.**

Heat-dried fecal slurries from two separate samples imaged under phase contrast microscope, with 100 µm scale bars based on imaging of a calibration slide under the same conditions (10X lens) shown. **A**, Example of small particles, and **B**, example of large particles.


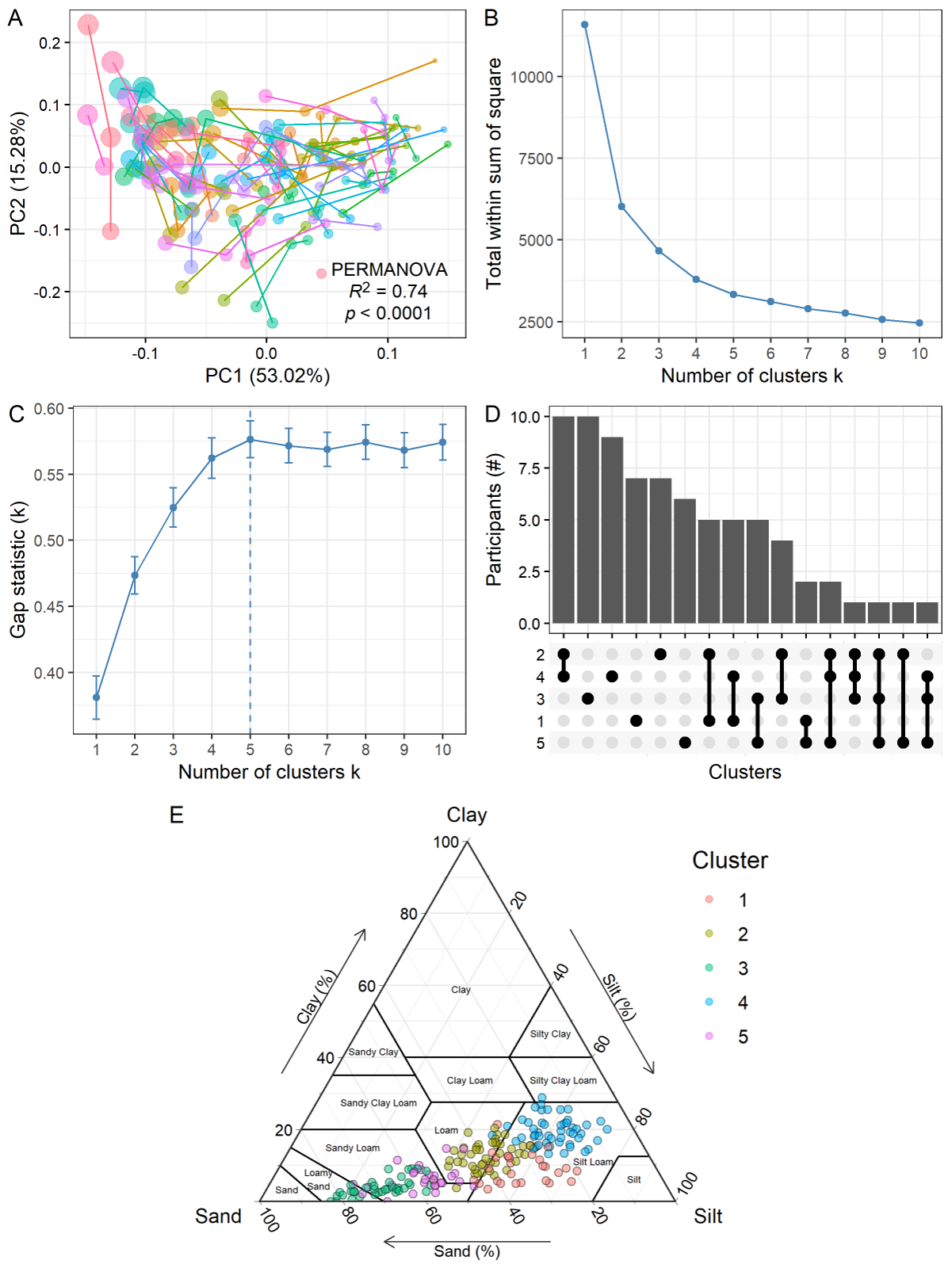


**Figure S4. Analysis of baseline particle size distributions.**

**A**, Principal component analysis of baseline sample particle size distributions, with points colored and connected by participant. Point size corresponds to median FPS for that sample. PERMANOVA statistic calculated on participant. (*n* = 186 samples from 76 participants; 41 in Cohort 1 and 35 in Cohort 2.) **B-C**, Total within sum of square (**B**) and gap statistic (**C**) plots used to determine the optimal number of clusters for k-means clustering. **D**, Upset plot showing the frequency of samples for each participant appearing in each cluster or combination of clusters (i.e. 10 participants had samples in both clusters 2 and 4, 10 participants had samples only in cluster 3, etc.). **E**, Ternary plot showing baseline samples on a USDA soil textural classification diagram, where sand is the largest particle size (50-2000 µm), silt is intermediate (2-50 µm), and clay is the smallest (< 2 µm).


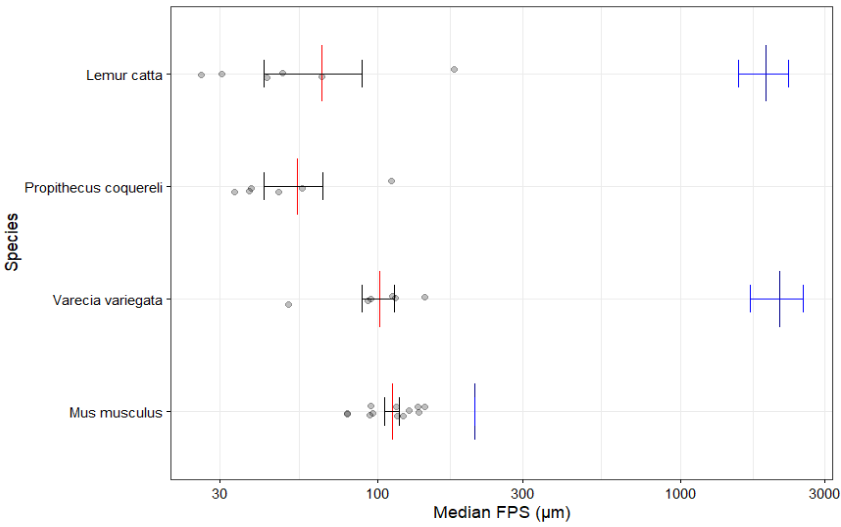


**Figure S5. Comparison of measured FPS to previously reported values across species.**

Median FPS shown on log scale for three species of lemur: *Lemur catta* (*n* = 6), *Propithecus coquereli*, (*n* = 6), and *Varecia variegata* (*n* = 6), as well as specific pathogen-free (SPF) mice (*n* = 10). Previously reported values from Fritz et al. (*J. Anim. Physiol. Anim. Nutr.*, 2009) mean and standard error shown (*Lemur catta* *n* = 3, *Propithecus coquereli* no data, *Varecia variegata n* = 4, and *Mus musculus* *n* = 1).

|  |
| --- |


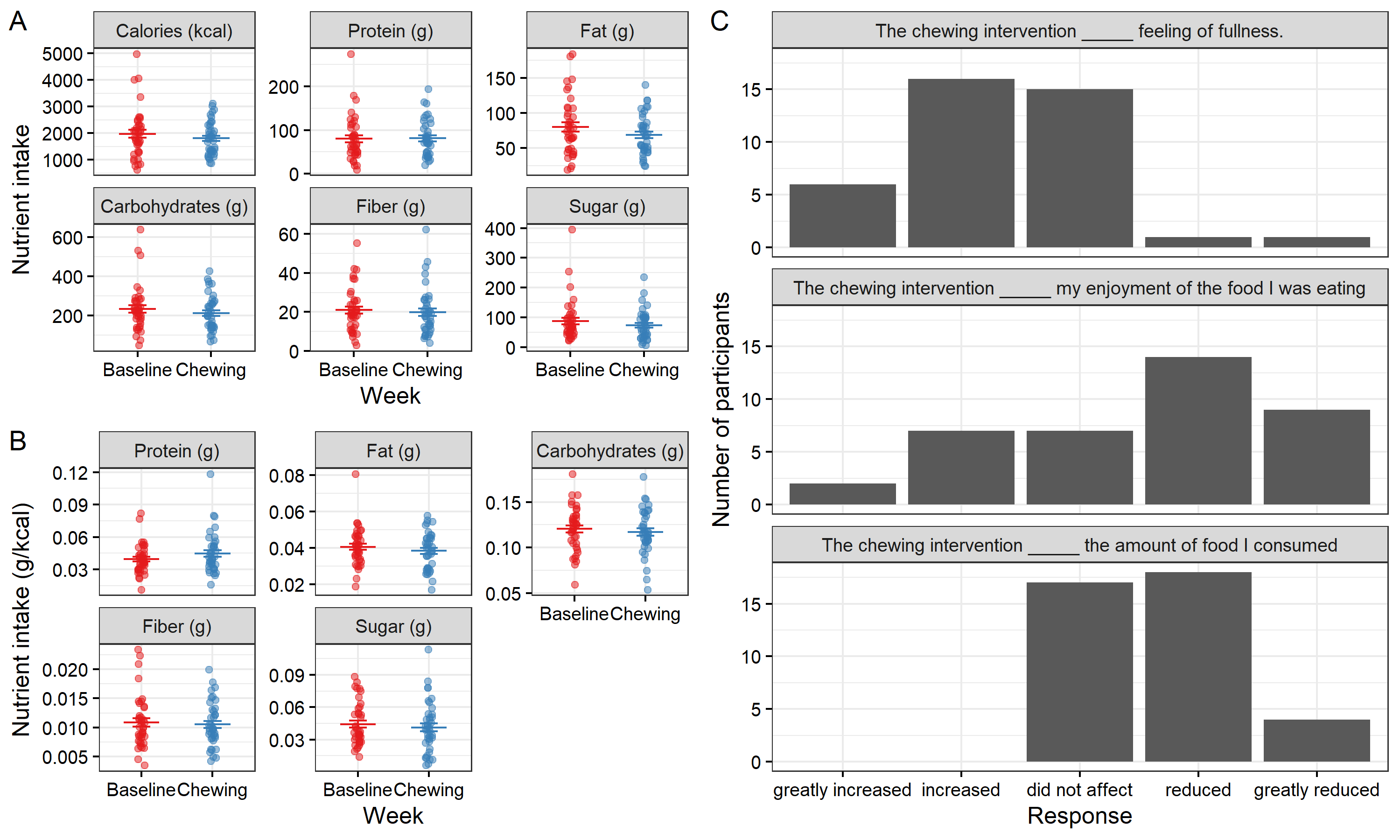


**Figure S6. Chewing study diet and exit survey data.**

**A-B**, Macronutrient intake reported on ASA24 by week in terms of raw values (**A**) or proportions relative to total kcal (**B**). Mean and standard error plotted. Linear mixed model (week as fixed effect, participant as random effect) *P* > 0.05 in all cases. **C**, Frequencies of responses to exit survey questions about chewing intervention. **A-C**, (*n* = 39 participants).

| 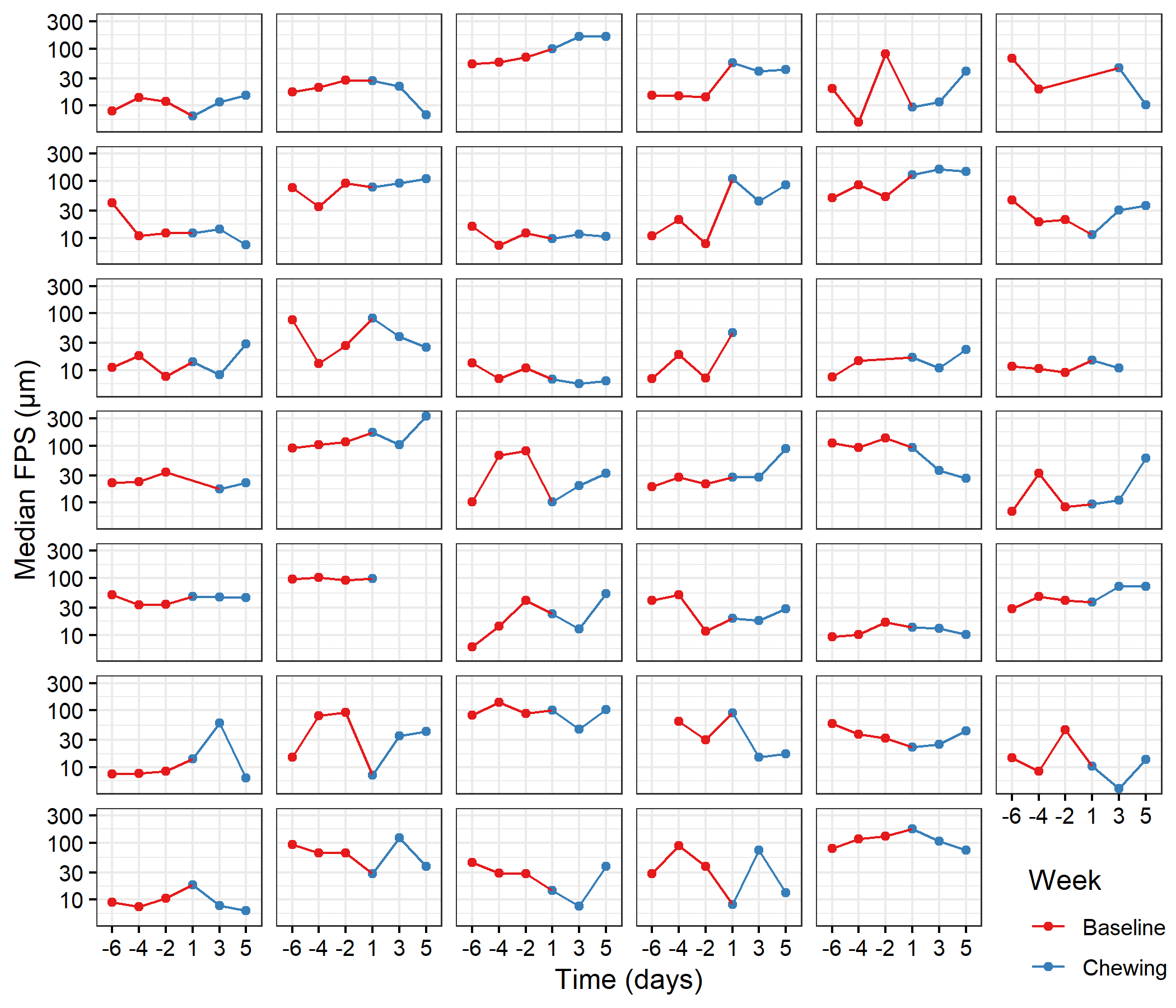 |
| --- |
| **Figure S7. Trends in median FPS by individual.**  Plots of median FPS over time for each individual in Cohort 1, where red points are from the baseline week and blue points are from the chewing week. |

| 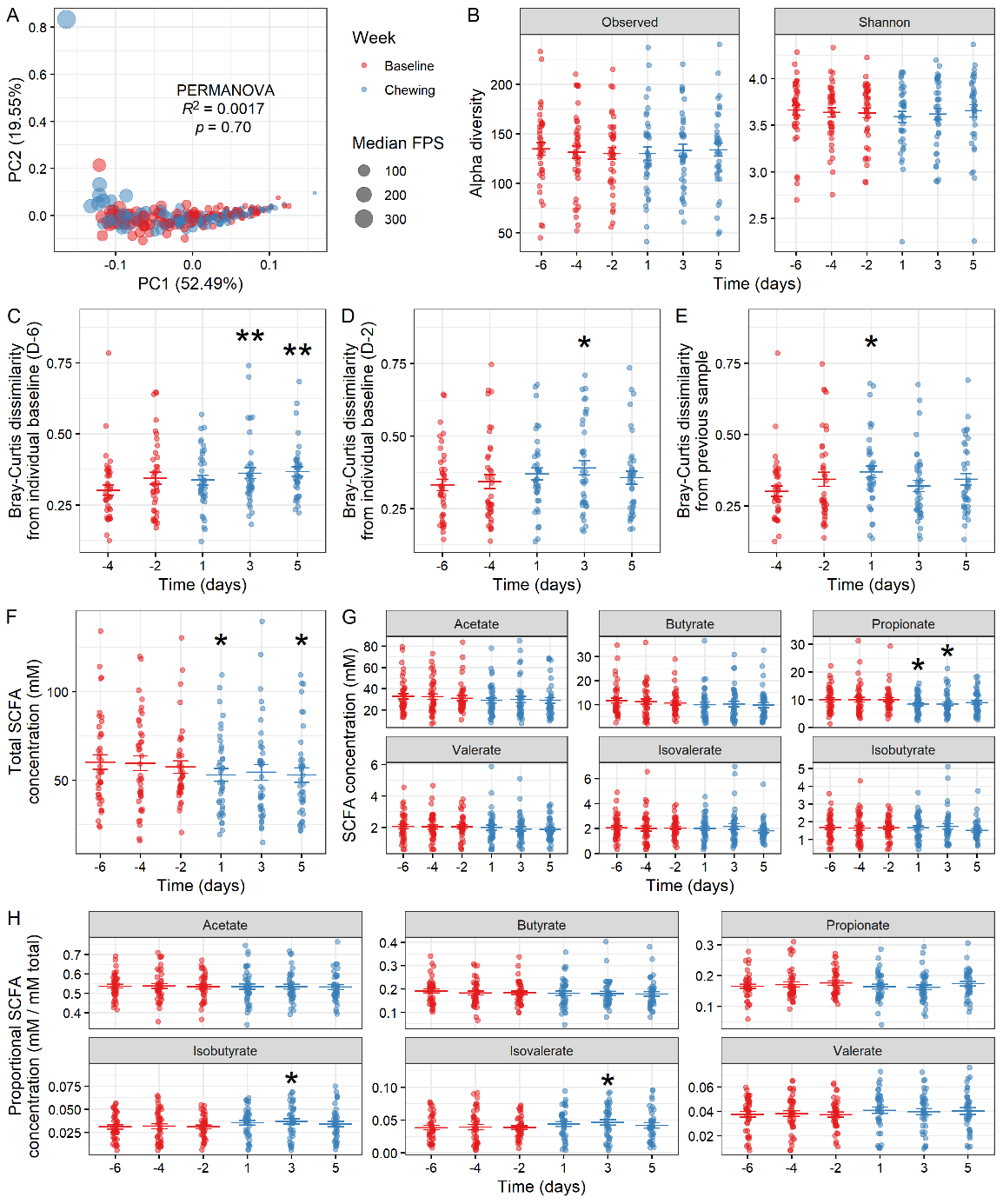 |
| --- |
| **Figure S8. Chewing study microbiome composition and SCFA data.**  **A**, PCA plot of particle size distributions by week, with PERMANOVA on week (participant as strata) shown. (*n* = 236 samples from 41 participants). **B**, Alpha diversity by observed ASVs and Shannon index by time point. Linear mixed model (day as fixed effect, participant as random effect), with day -6 as intercept, *P* > 0.05; for week as fixed effect, *P* = 0.53 for observed ASVs and *P* = 0.21 for Shannon. **C-E**, Bray-Curtis dissimilarity relative to each participant’s first sample provided (**C**), the day -2 sample (**D**), or the previous sample (**E**). Linear mixed model (day as fixed effect, participant as random effect), with earliest time point as intercept, shown; for week as fixed effect, *P* = 0.026 (**C**), *P* = 0.0133 (**D**), and *P* = 0.17 (**E**). **F-H**, Total (**F**) and individual (**G**) SCFA concentrations or proportions (**H**) by time point. Linear mixed models (day as categorical fixed effect, participant as random effect), with day -6 as intercept, shown; for week as fixed effect, *P* < 0.05 for acetate, propionate, and valerate concentrations (**F**), and *P* < 0.05 for isobutyrate and isovalerate proportions (**H**). **B-H**, Mean and standard error plotted. (*n* = 41 participants). * *P* < 0.05, ** *P* < 0.01, *** *P* < 0.001. |

| 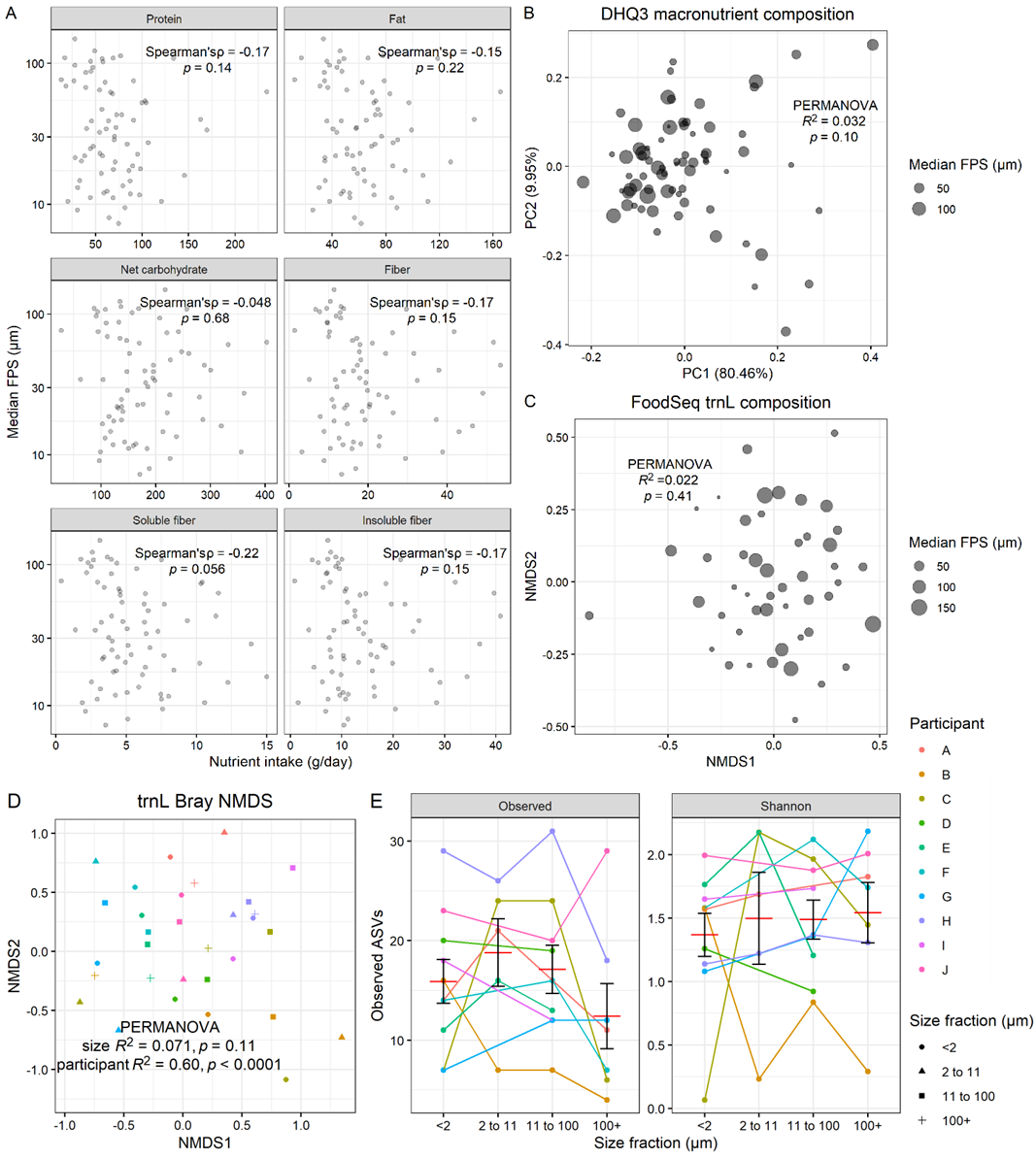 |
| --- |
| **Figure S9. Relationship of FPS with diet.**  **A**, Relationship between habitual intake of macronutrients as reported by DHQ3, with a focus on dietary fiber, and median FPS. Results of Spearman correlation test shown. **B**, Data from (**A**) ordinated onto PCA space, with results of PERMANOVA by median FPS shown. **A-B**, (*n* = 73 participants.) **C,** NMDS plot of food DNA by trnL sequencing using FoodSeq, with PERMANOVA by median FPS with participant as strata shown (Cohort 2 only; *n* = 35 participants). **D-E**, Analysis of trnL diet metabarcoding sequencing of *n* = 10 fractionated stool samples. **D**, NMDS ordination plot. Results of PERMANOVA (size + participant) shown. **E**, trnL alpha diversity by particle size fraction. ANOVA (size + participant) for Observed: size *P* = 0.054, participant *P* = 0.22; for Shannon: size *P* = 0.13, donor *P* = 0.95. |
| 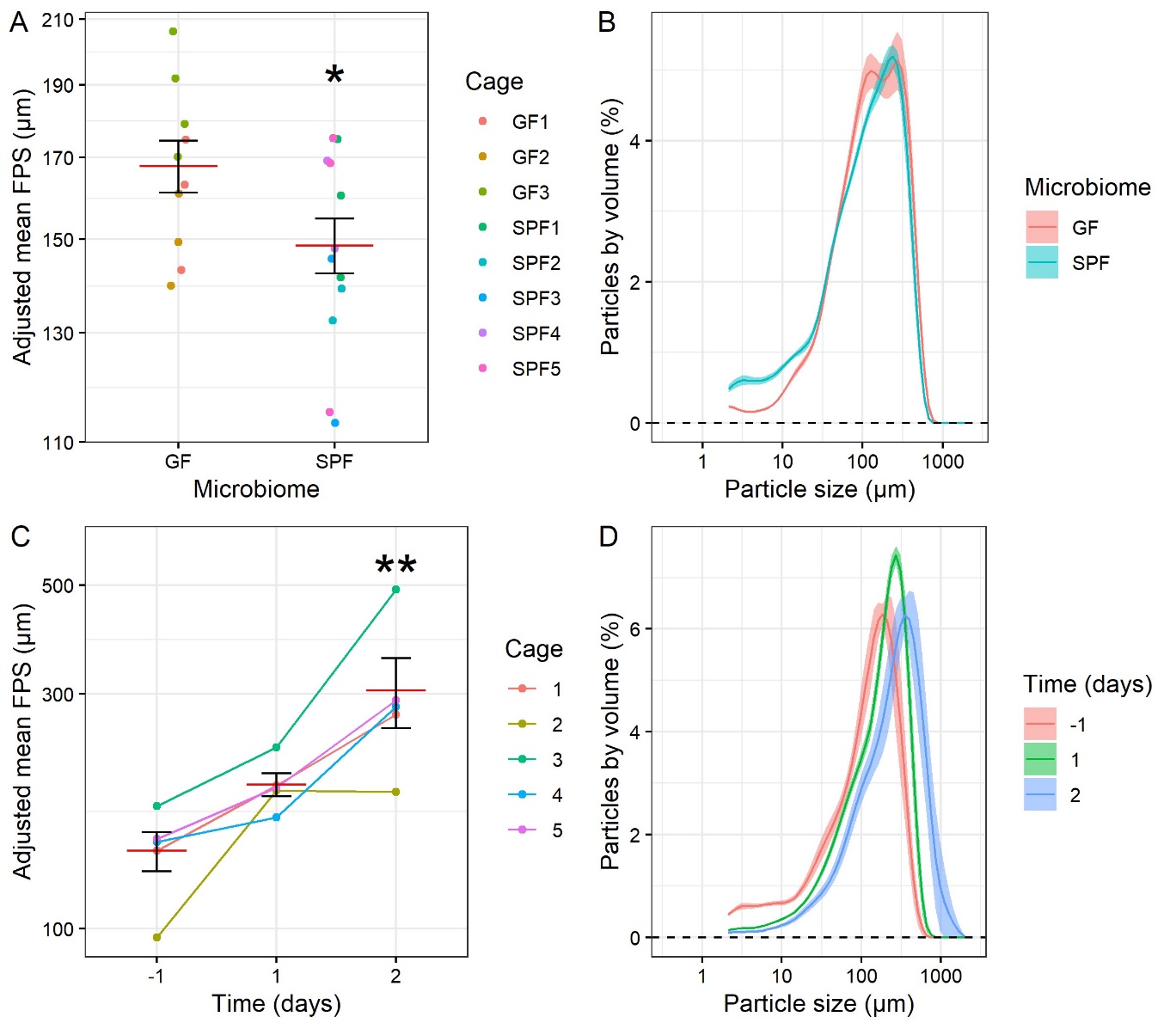 |
| **Figure S10. Re-analysis of mouse samples with exclusion of bacteria-range particles.**  Data for mouse experiments shown in Fig. 3, recomputed to omit particles < 2 µm. **A**, Mean FPS in germ-free (GF) and specific-pathogen-free (SPF) mice collected from individual mice. (Linear model with GF as intercept; *n* = 10 GF mice and 12 SPF mice.) **B**, Particle size distributions for (**A**). **C**, Mean FPS for mouse fecal samples collected by cage, with imipenem antibiotic treatment begun on day 0. Linear mixed model (day as categorical fixed effect, cage as random effect) with D-1 as intercept shown. (*n* = 5 cages.) **D**, Particle size distributions for (**C**). **A-E**, Mean and standard error plotted. * *P* < 0.05, ** *P* < 0.01, *** *P* < 0.001. |

| **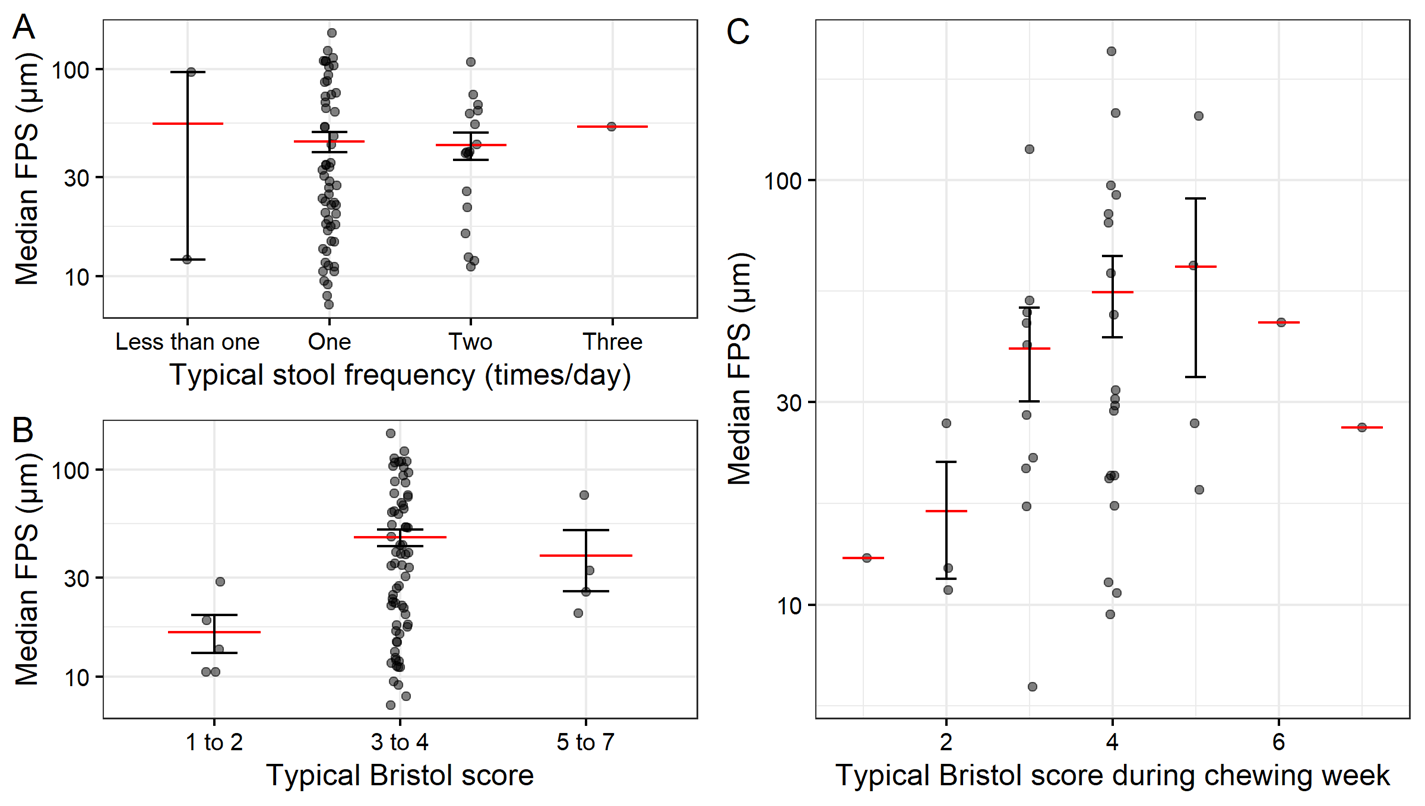** |
| --- |
| **Figure S11. Relationships of FPS with self-reported stool statistics.**  **A-B**, Self-reported typical stooling frequencies **(A)** and Bristol stool scores **(B**) plotted against average median FPS for each participant. ANOVA *P* > 0.05 for both variables (*P* = 0.15 for Bristol score and *P* = 0.97 for frequency; *n* = 76 participants.) **C,** Exit survey responses to the question “What type of stool did you most often experience over the past week?” in Cohort 1, plotted against the average median FPS within that week for each participant. (*n* = 39 participants.) **A-C,** Mean and standard error plotted. |

| **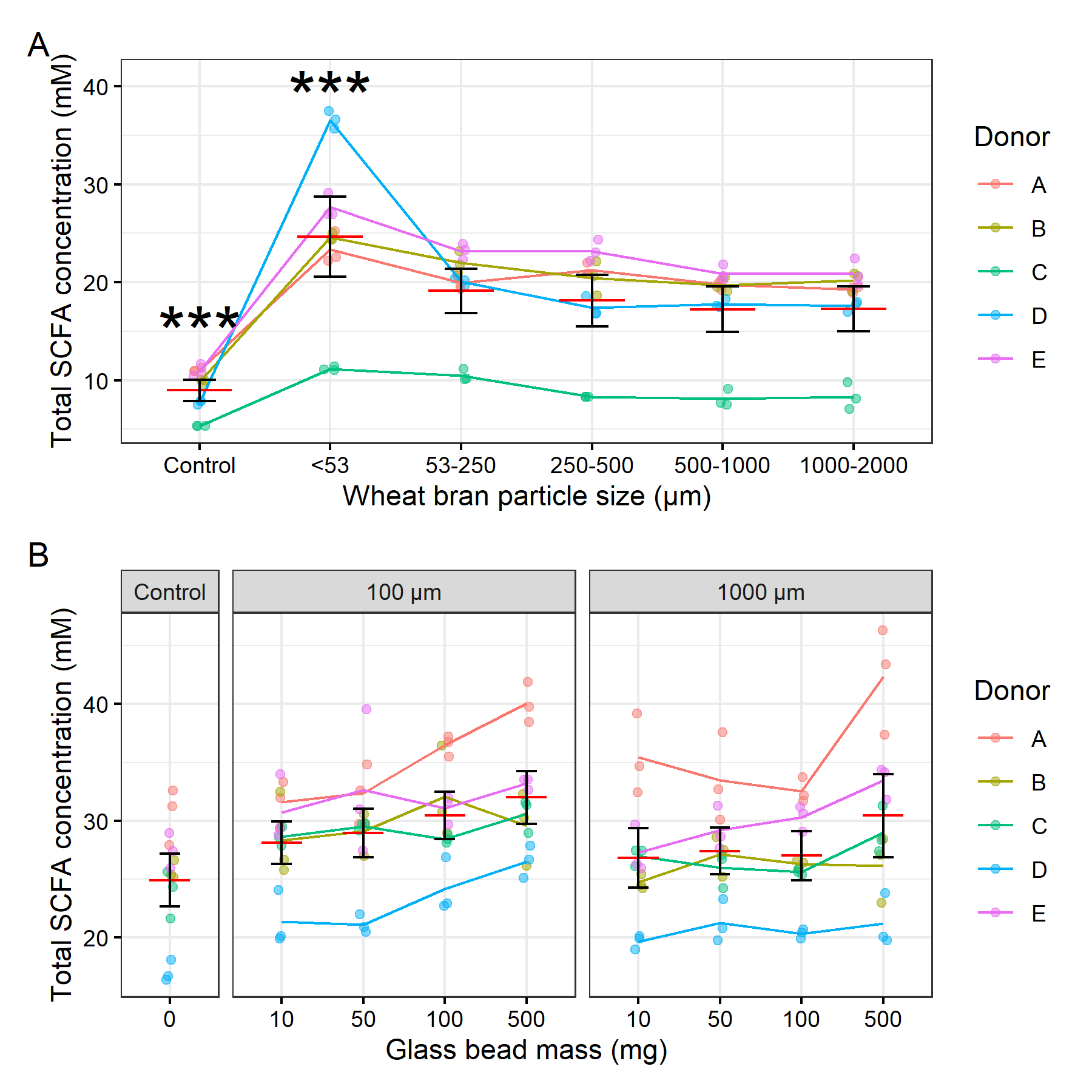** |
| --- |
| **Figure S12. Substrate particle size influences gut microbial metabolism *in vitro*.**  **A,** Total SCFA concentration after 24-hour incubation of stool-derived microbial communities with wheat bran particles of different sizes. Linear mixed model (size as fixed effect, donor as random effect) with 1000-2000 as intercept shown. (*n* = 5 donors with 3 technical replicates each.) * *P* < 0.05, ** *P* < 0.01, *** *P* < 0.001. **B,** Total SCFA concentration after 24-hour incubation of stool-derived microbial communities with small or large glass beads added at different masses. Linear mixed model (size:mass as fixed effects, donor as random effect), excluding control, with 100 µm glass beads and 10 mg bead mass as intercept conditions, gives size *P* = 0.0014, mass *P* = 0.00013, size:mass *P* = 0.83. (*n* = 5 donors with 3 technical replicates each.) **A-B**, Mean and standard error plotted. |

| 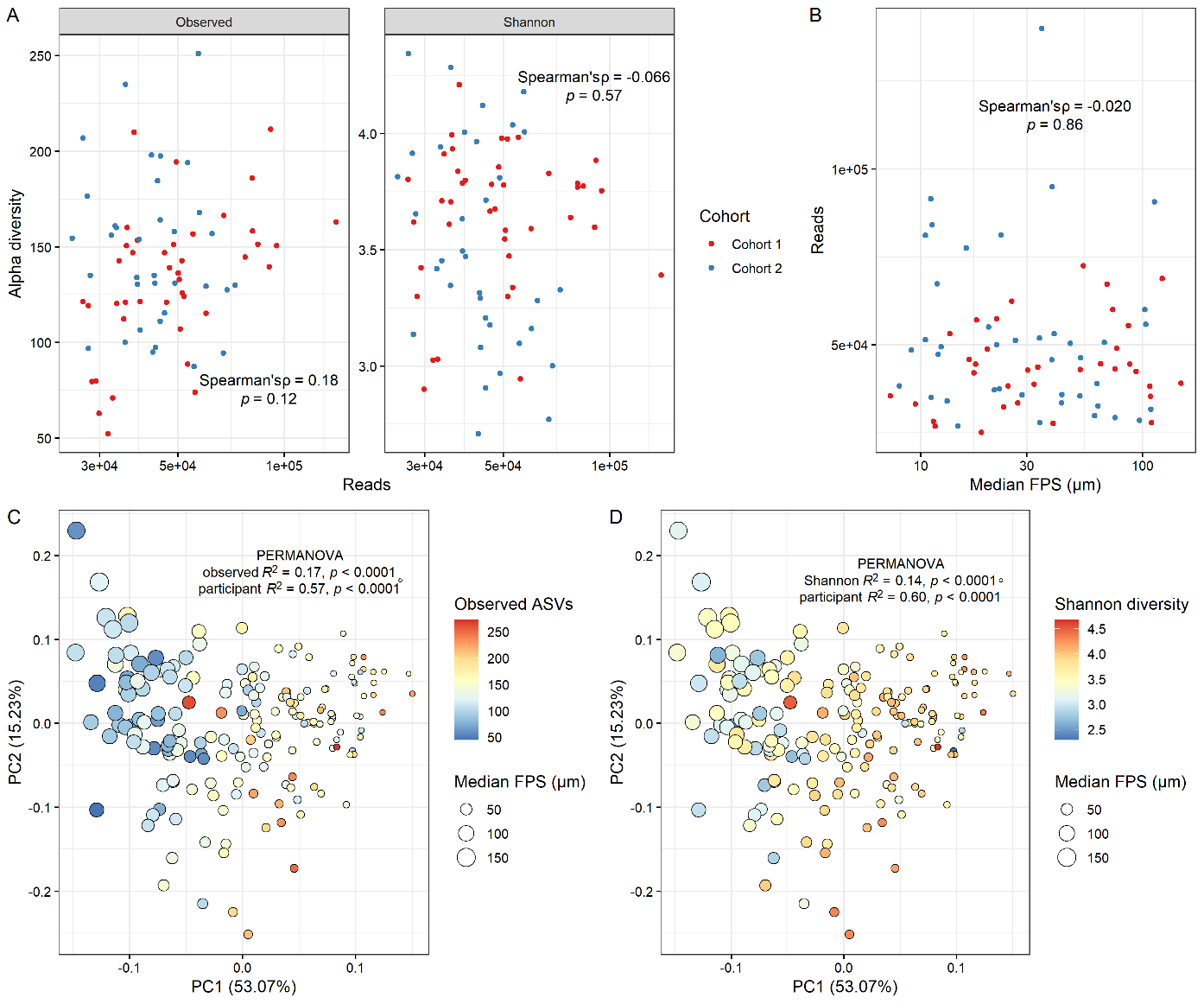 |
| --- |
| **Figure S13. FPS and alpha diversity.**  **A**, Scatterplot of read depth averaged within each participant and alpha diversity by observed ASVs and Shannon index. Spearman correlation test statistics shown. (*n* = 76 participants; 41 in Cohort 1 and 35 in Cohort 2.) **B**, Scatterplot of median FPS averaged within each participant and read depth. Spearman correlation test statistics shown. **C-D**, PCA of particle size distributions colored by alpha diversity using observed ASVs (**C**) or Shannon index (**D**), with results of PERMANOVA (diversity + participant) shown. (*n* = 185 samples.) |

| 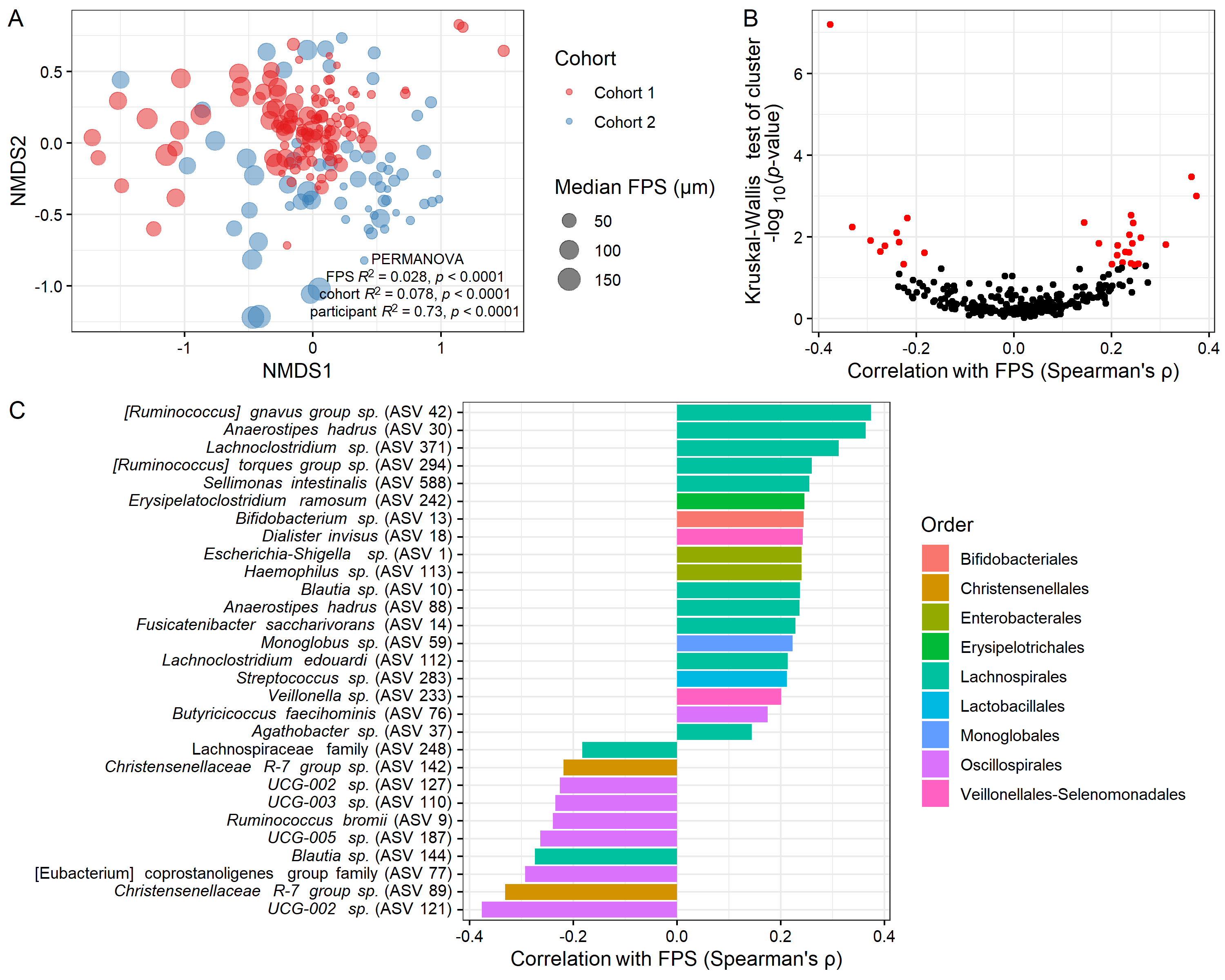 |
| --- |
| **Figure S14. FPS and community composition.**  **A**, NMDS ordination plot equivalent to Fig. 1C, colored by cohort, with results of PERMANOVA (cluster + cohort + participant) shown. (*n* = 185 samples.) **B**, Volcano plot depicting results of ALDEx2 Kruskal-Wallis test on particle size distribution cluster as a categorical variable, with ASVs found to be significantly differentially abundant by cluster (FDR-corrected *P* < 0.05) shown in red. Each point is an ASV (298 tested, 29 significant), and each ASV’s Spearman correlation with FPS is shown on the x-axis as an effect size metric. **C**, Identities of ASVs found to be significantly differentially abundant across the five clusters (red points in (**B**)), plotted against the Spearman correlation coefficient and colored by order. |

| 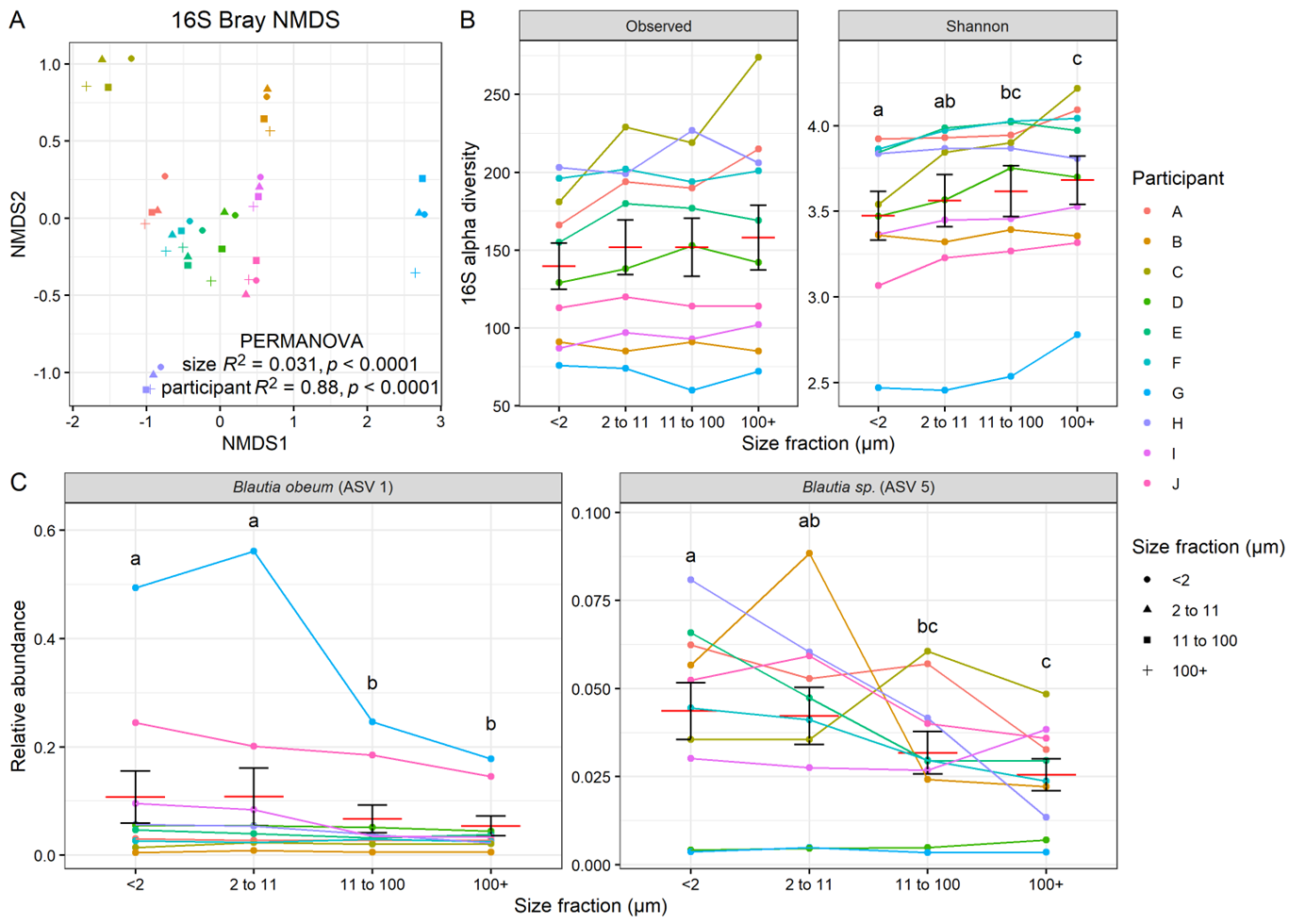 |
| --- |
| **Figure S15. 16S rRNA sequencing of particle fractions of ten samples.**  **A**, NMDS ordination plot of 16S rRNA gene amplicon sequencing of ten fractionated stool samples from different participants. Results of PERMANOVA (size + participant) shown. **B**, 16S alpha diversity by particle size fraction. ANOVA (size + participant) for Observed: size *P* = 0.050, participant *P* = 8.7 × 10^-16^; for Shannon: size *P* = 0.00025, donor *P* < 2 × 10^-16^; letters indicate significantly different (*P* < 0.05) groups by Tukey HSD test. **C**, We identified two ASVs as significantly different by size fraction by ALDEx2 GLM (size + participant); letters indicate significantly different (*P* < 0.05) groups by Tukey HSD test. **A-C**, (*n* = 10 participants.) |


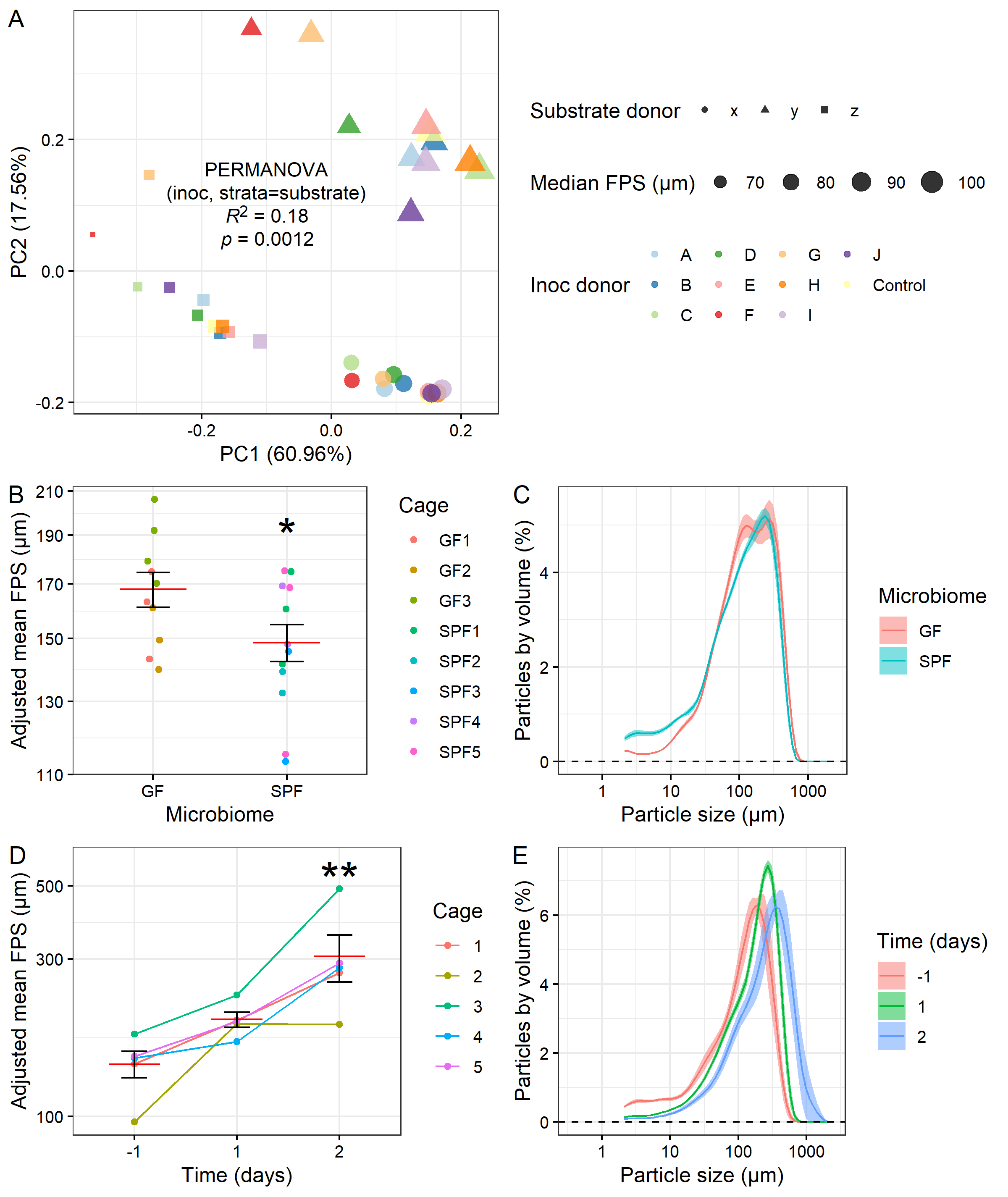

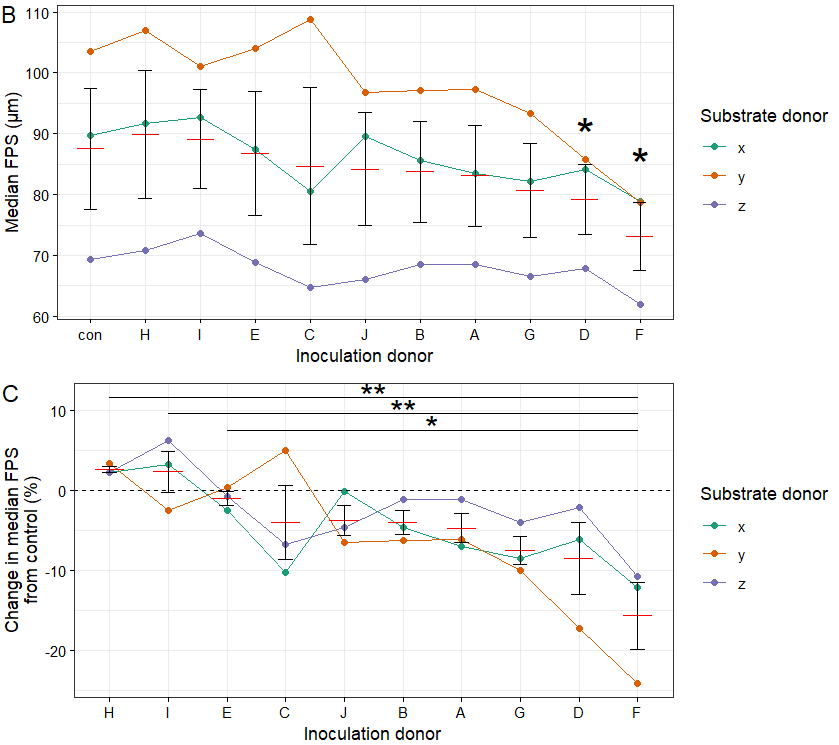


**Figure S16. Cross-inoculation experiment.**

**A**, PCA plot of post-incubation particle size distributions for *ex vivo* cross-inoculation experiment. Stool samples from three donors were disinfected by incubation in ethanol, then inoculated with overnight cultures from one of additional stool donors, or no-inoculation control. Result of PERMANOVA on inoculation donor with substrate donor as strata shown. In general, the three substrate donors (shapes) cluster separately; within those, the relative positions of inoculation donors (colors) are generally consistent. **B**, Median FPS by inoculation donor, with points and lines colored by substrate donor. Results of linear mixed model compared to control shown (inoculation donor as fixed effect, substrate as random effect). **C,** Data from (**B**), re-calculated as percent change in median FPS from control (for each substrate) in order to account for the significant variation in substrate particle size. ANOVA (cluster + participant) *P* = 0.0060 for inoculation donor and *P* = 0.17 for substrate donor. Results of Tukey HSD test shown on plot. **B-C**, Mean and standard error plotted. * *P* < 0.05, ** *P* < 0.01, *** *P* < 0.001.
